# Supplementary material for: Hospital inpatient care utilization among patients with tuberculosis, Republic of Ireland, 2015–2018
Source: PLoS One. 2020 Aug 27;15(8):e0238142. doi: 10.1371/journal.pone.0238142 (PMC7451637; doi:10.1371/journal.pone.0238142)
Supplement: S1 File — (DOCX) [file pone.0238142.s001.docx]

Table S1: Categorical Data Analysis

| Comparison of Patient Subgroups by Age | | | |  |  |
| --- | --- | --- | --- | --- | --- |
|  | Mean | Std. Err. | 95% Conf. lower limit | 95% Conf. upper  Limit | P Value |
| Patients without SDRFs for TB | 43.09 | 0.79 | 41.55 | 44.64 | 0.000 |
| Patients with SDRFs for TB | 52.18 | 1.31 | 49.61 | 54.75 |  |
|  |  |  |  |  |  |
| Association between Gender and having a SDRF for TB | | | | |  |
|  | Female | Male | Total |  |  |
| No SDRFs for TB | 290 | 390 | 680 |  |  |
| SDRFs for TB | 29 | 109 | 138 |  |  |
| Total | 319 | 499 | 818 |  |  |
| Pearson chi2(1) = 22.566 |  |  |  |  | 0.000 |
|  |  |  |  |  |  |
| Association between TB disease site and having a SDRF for TB | | | |  |  |
|  | Non-respiratory TB | Respiratory TB | Total |  |  |
| No SDRFs for TB | 180 | 500 | 680 |  |  |
| SDRFs for TB | 23 | 115 | 138 |  |  |
| Total | 203 | 615 | 818 |  |  |
| Pearson chi2(1) =5.9098 |  |  |  |  | 0.02 |

Table S2 Comparison of Cost of Episodes of Care in Patients with and without Social Determinants and Risk Factors for TB

|  | SDRFs for TB | | | No SDRFs for TB | | |
| --- | --- | --- | --- | --- | --- | --- |
|  | Emergency episodes of care | Elective episodes of care | All | Emergency episodes of care | Elective episodes of care | All episodes of care |
| Total (proportion of all) | 164  (79.6%) | 42  (20.4%) | 206 | 637  (65.1%) | 342  (34.9%) | 979 |
| Number of patients  (proportion of all) | 124  (89.8%) | 35  (25.4%) | 138 | 515  (75.7%) | 252  (37%) | 680 |
| Number of bed-days 2015-2018 | 4751 | 508.5 | 5259.5 | 9309.5 | 1436 | 10745.5 |
| Median length of stay (days) (IQR) | 15.5  (7-31.25) | 2  (0.5-11.25) | 14  (6-29) | 9  (4-17) | 0.5  (0.5-2) | 6  (0.5-14) |
|  | Cost (lower-upper limit) | Cost (lower-upper limit) | Cost (lower-upper limit) | Cost (lower-upper limit) | Cost (lower-upper limit) | Cost (lower-upper limit) |
| Total cost (€) | 2,702,921- 2,963,567 | 405,328-449,427 | 3,152,348- 3,368,895 | 6,300,781- 7,266,020 | 1,102,183-1,185,271 | 7,402,964-8,451,291 |
| Mean cost per year (€) | 675,580-740,892 | 101,332-112,357 | 788,087-842,224 | 1,575,195-1,816,505 | 275,546-296,318 | 1,850,741-2,112,823 |
| Mean cost per episode (€) | 16,481- 18,071 | 2,851-  4,091 | 3,826- 4,088 | 9,891- 11,407 | 666- 679 | 1,890- 2,158 |
| Mean cost per bed-day (€) | 569- 624 | 797- 884 | 559- 641 | 677- 780 | 767- 825 | 689- 786 |

Table S3 Comparison of First Emergency Episode of Care in Patients with Respiratory TB and Non-respiratory TB

| 1^st^ Emergency Episode of Care | Respiratory TB | Non-respiratory TB | Odds Ratio*  (95% Confidence Interval) | P Value |
| --- | --- | --- | --- | --- |
| Median length of stay (days) (IQR) | 10 (5-19) | 13 (6-21) | .994  (.987-1.001) | .111 |
| Median cost per thousand (€) (IQR)  (Minimal complexity assumed where complexity level unknown) | 5.9 (5.6-5.9) | 7.9 (5.9-17.6) | .978  (.966-.991) | .001 |
| Median cost per thousand (€) (IQR)  (Major complexity assumed where complexity level unknown) | 10.7 (4.7-10.7) | 9.8 (6.3-17.6) | .984  (.972-.995) | 0.005 |

*Odds ratio derived from univariate logistic regression

Table S4 Comparison of First Emergency Episode of Care in Patients with SDRFs for TB and Patients without SDRFs for TB

| 1^st^ Emergency Episode of Care | SDRFs for TB | No SDRFs for TB | Odds Ratio*  (95% Confidence Interval) | P Value |
| --- | --- | --- | --- | --- |
| Median length of stay (IQR) | 16 (8-33) | 9(4-17) | 1.017  (1.009-1.025) | .000 |
| Median cost per thousand (€) (IQR)  (Minimal complexity assumed where complexity level unknown) | 15.9 (5.9-13.2) | 5.9 (5.3-7) | 1.01  (1.001- 1.019) | .034 |
| Median cost per thousand (€) (IQR)  (Major complexity assumed where complexity level unknown) | 10.7(10.6-12.5) | 10.7 (4.7-10.7) | 1.011  (1.001-1.021) | .026 |

*Odds ratio derived from univariate logistic regression

Table S5 Comparison of First Emergency Episode of Care in Patients with a Charlson Comorbidity Index of Zero and Patients with a Charlson Comorbidity Index Greater than 0

| 1^st^ Emergency Episode of Care | Charlson Comorbidity Index=0 | Charlson Comorbidity Index>0 | Odds Ratio*  (95% Confidence Interval) | P Value |
| --- | --- | --- | --- | --- |
| Median length of stay (IQR) | 9 (4-17) | 19.5 (10-34) | 1.024  (1.015-1.033) | .000 |
| Median cost per thousand (€) (IQR)  (Minimal complexity assumed where complexity level unknown) | 5.9 (5.1-6.4) | 7.9 (5.9-19.5) | 1.045  (1.028-1.062) | .000 |
| Median cost per thousand (€) (IQR)  (Major complexity assumed where complexity level unknown) | 10.7 (4.7-10.7) | 10.7 (10.7-19.1) | 1.05  (1.032- 1.069) | .000 |

*Odds ratio derived from univariate logistic regression

Table S6 Comparison of First Emergency Episode of Care in Patients with Drug Resistant TB and Patients with Drug Sensitive TB

| 1^st^ Emergency Episode of Care | Drug Resistant Tuberculosis | Drug Sensitive Tuberculosis | Odds Ratio*  (95% Confidence Interval) | P Value |
| --- | --- | --- | --- | --- |
| Median length of stay (IQR) | 30 (7-55) | 10 (5-20) | 1.016  (1.004-1.027) | .004 |
| Median cost per thousand (€) (IQR)  (Minimal complexity assumed where complexity level unknown) | 13.1 (5.9-20.1) | 5.9 (5.9-7.9) | 1.009  (.994- 1.023) | .233 |
| Median cost per thousand (€) (IQR)  (Major complexity assumed where complexity level unknown) | 13.7 (8.4-18.2) | 4.7 (4.7-10.7) | 1.007  (.991-1.02) | .378 |

*Odds ratio derived from univariate logistic regression
