# Supplementary material for: Hospital inpatient care utilization among patients with tuberculosis, Republic of Ireland, 2015–2018
Source: PLoS One. 2020 Aug 27;15(8):e0238142. doi: 10.1371/journal.pone.0238142 (PMC7451637; doi:10.1371/journal.pone.0238142)
Supplement: Attachment — Submitted filename: Response to reviewers 2nd revision.docx [file pone.0238142.s003.docx]

Date Typed 03/08/2020

Dear Editor and Reviewers,

Thank you for once more taking the time to review our submission and providing rigorous feedback. Our responses to the comments are outlined below. We hope the changes submitted are sufficient to meet the standard of publication.

1*. “There were unfortunate limitations to the datasets used by the authors. It is suggested that, in the Discussion, they mention this limitation and that efforts be made to address it, so that in the future they can track, yet more completely, the effect of their reforms.”*

Yes, we agree this is the prime limitation in the research method. We have included a comment which highlights this limitation. We feel that our matching process based on age, gender, county of residence and postal code could potentially match episodes of care to the same patient when in fact they may have been due to different patients. This means the number of patients in the dataset is, if anything, an underestimation meaning the problem highlighted of a high proportion of cases notified requiring hospitalization may even be higher than estimated in this paper. We highlight an already ongoing project in the Republic of Ireland to provide a unique patient identifier across all public hospitals which would overcome this problem when evaluating the service in the future.

The second notable limitation is that the hospital coding data collection process in the ROI is imperfect and lacks specificity i.e. when the coder was uncertain of how to code an episode of care they allocate it to a non-specific ICD-10 code and they may allocate a diagnosis related grouping of lower complexity and therefore lower cost. We cite a national audit which found the primary reason for these discrepancies is that coders were unable to find the relevant information in poorly maintained hospital paper records. We highlight an ongoing national project to rollout an electronic health record to all public hospitals.

2. *“although in the text, the authors present evidence of statistical significance through the use of p-values, these are not presented in the tables at all. I strongly suggest that they be presented in a column in the tables; standard practice, as far as I know”*

We have included a column labelled “comparison” which includes the comparison made and the associated p-value. In the supplementary file a column for P values is also included in each table.

3. *“One of the assumptions of the Mann-Whitney U test is independence of observations. If you have 800 or so people but 1180 episodes of care, at least some of those are going to be on the same individuals, and thus may violate this assumption. You may want to include a sentence in your limitations section about that”*

We agree with this statement. We have re-analysed our data. We have performed logistics regression instead of a Mann-Whitney U test to compare length of stay and cost of episodes of care between patients with respiratory and non-respiratory TB, patients with SDRFs for TB and those without SDRFs for TB and those with comorbidity and those without comorbidity (as measured by the Charlson Comorbidity Index). We have performed this analysis for all patients first episode of care to ensure there is independence in the observations analysed. We have included the results of these analyses, including p-values, in the supplementary file.

4.*“line 229: unbalanced number of parentheses”*

We have corrected this.

5.*“Table 2, respiratory TB/Elective box unbalanced parentheses”*

We have corrected this.

6.*“line 263 and beyond--ensure you are using a "chi" symbol, and not an "X"”*

We have corrected this.

7.*“Table 6, no SDRFs columns are missing some percentages”*

We have corrected this.

Yours Sincerely,

James O’Connell
